# Hormonal Atrial Fibrillation: Pathophysiological Mechanisms That Trigger and Sustain the Arrhythmic Circuits

**DOI:** 10.3390/biomedicines13102466

**Published:** 2025-10-10

**Authors:** Letizia Rosa Romano, Aldo Celeste, Antonio Curcio

**Affiliations:** 1 Coronary Care Unit, Division of Cardiology, Azienda Ospedaliera di Cosenza, 87100 Cosenza, Italy; 2 School of Cardiovascular and Metabolic Health, University of Glasgow, Glasgow G12 8QQ, UK; 3 Department of Pharmacy, Health and Nutritional Sciences, University of Calabria, 87036 Rende, Italy

**Keywords:** arrhythmia, pathophysiology, molecular mechanisms, sex-specific cardiovascular outcomes, precision medicine

## Abstract

Atrial fibrillation (AF) is the supraventricular tachy-arrhythmia most commonly detected in the general population, with significant sex-related differences in epidemiology, pathophysiology, and treatment outcomes. Emerging evidence highlights the role of sex hormones—particularly estrogen and testosterone—in modulating left atrial electrophysiologic substrate, structural remodeling, inflammation, and thromboembolic risk. Hormonal fluctuations across different lifespan influence AF onset, progression, and therapeutic response, yet current management approaches largely overlook such determinants. This narrative review integrates data from basic, translational, and clinical research to examine hormonal effects on atrial substrate, disease progression, and differential results of treatments, including stroke prevention, pharmacological options, and transcatheter ablation. It also explores the potential of hormone-targeted interventions, antifibrotic therapies, and precision strategies tailored to hormonal status. Addressing these mechanisms could optimize patient-specific management, improve outcomes and guide future clinical practice recommendations. Advancing toward sex-specific, hormone-informed AF care requires further mechanistic studies, hormonal profiling, and sex-stratified clinical trials.

## 1. Introduction

Atrial fibrillation (AF) is the most prevalent cardiac arrhythmia encountered in clinical practice, representing a major global health challenge due to its association with increased morbidity, mortality, and healthcare burden. Its prevalence is rising steadily, driven by population aging and the growing prevalence of cardiovascular (CV) comorbidities [1]. Despite significant advances in prevention, diagnosis, and treatment, AF remains a leading cause of stroke, heart failure (HF), and reduced quality of life (QoL). While sex-specific differences in AF epidemiology, symptom burden, and outcomes have long been recognized, the influence of sex hormones on atrial structure, function, and therapeutic response has only recently gained focused attention [2].

Emerging evidence suggests that hormonal status, particularly variations in estrogen and testosterone, plays a significant role in modulating atrial electrophysiology, structural remodeling, and thromboembolic risk. These endocrine influences contribute to the observed disparities between men and women in AF onset, progression, and treatment outcomes [3]. However, the translation of such mechanistic insights into tailored clinical strategies remains limited. This gap may contribute to suboptimal outcomes in the female gender, since it becomes affected later by the disease, with more advanced left atrial (LA) remodeling, and remains underrepresented in randomized controlled trials [4].

The clinical relevance of addressing this gap is substantial. A deeper understanding of how hormonal status shapes arrhythmia mechanisms could inform individualized therapy, optimize the timing and selection of rhythm- vs. rate-control strategies, refine ablation approaches, and improve stroke prevention measures. Furthermore, understanding how hormonal fluctuations affect various cardiac cellular populations may help identify novel therapeutic targets, including antifibrotic, anti-inflammatory, and metabolic interventions, as well as guide the safe and effective use of hormone replacement therapy (HRT) in selected populations [5].

To support this narrative review, we performed a non-systematic literature search in PubMed and Scopus from 2000 to 2024 using the terms ‘atrial fibrillation’, ‘sex hormones’, ‘estrogen’, ‘progesterone’, ‘testosterone’, and ‘atrial remodeling’. Additional relevant articles were identified through reference lists. Priority was given to original studies, large registries, and recent reviews. No formal inclusion or exclusion criteria were applied beyond relevance to the topic.

The primary aim is to synthesize current knowledge on the interplay between sex hormones and AF, integrating evidence from basic science, translational research, and clinical studies. Through an in-depth examination of hormonal influences on atrial remodeling and treatment responsiveness, this work attempts to uncover opportunities for tailored, sex-specific therapeutic strategies. In this context, it aims to bridge the translational divide between pathophysiological evidence and clinical practice, providing researchers and clinicians with a coherent framework to guide further investigations.

## 2. Sex Hormones in Regulating Cardiac Structure and Function

Beyond their classical roles in reproductive biology, estrogens, androgens, and progesterone participate actively in the maintenance of cardiac homeostasis through both genomic and non-genomic mechanisms. These effects are mediated via the activation of specific intracellular and transmembrane receptors, which are differentially expressed in cardiomyocytes, fibroblasts, endothelial cells, vascular smooth muscle cells, components of the cardiac conduction system, and cardiac pericytes [6,7,8].

Estrogens, primarily estradiol (E2), exert cardioprotective effects mediated by α and β estrogen receptors (ER-α and ER-β), which are expressed in both cardiomyocytes and fibroblasts. These receptors regulate gene transcription associated with antioxidant defense, nitric oxide (NO) production, calcium handling, and anti-fibrotic pathways. ER-β, in particular, has been associated with protective remodeling and anti-inflammatory effects in atrial tissue [9]. The hormonal milieu is dynamic and undergoes substantial shifts throughout life. In women, cyclic variations during the menstrual cycle result in fluctuations of estrogen and progesterone, which transiently modulate cardiac electrophysiology and autonomic tone, as demonstrated by phase-dependent alterations in heart rate variability, sympathetic outflow, and baroreflex sensitivity [10]. During menopause, the sharp decline in circulating estrogens disrupts hormonal equilibrium and removes estrogen-related cardioprotective effects, leading to increased CV vulnerability via endothelial dysfunction and a documented rise in CV risk following early menopause [11,12]. HRT in postmenopausal women, while intended to mitigate the effects of estrogen loss, has shown variable influence on cardiac structure and arrhythmic risk depending on the formulation, dosage, and timing of initiation [13,14].

Under physiological conditions, androgen receptors (AR), which bind testosterone and its active metabolite dihydrotestosterone, are expressed throughout the myocardium and regulate of cardiac excitability, pro-hypertrophic signaling pathways, and extracellular matrix (ECM) remodeling. The expression levels, distribution, and functional activity of AR are dynamically modulated by circulating androgen concentrations and exhibit significant variation according to sex, developmental stage, and physiological or pathological context [15].

In men, testosterone levels gradually decline with age, a process often referred to as late-onset hypogonadism. This condition has been associated with an increased incidence of cardiovascular events and arrhythmias. Low testosterone is also linked to enhanced inflammation, endothelial dysfunction, and myocardial fibrosis [16,17].

Although less common, supraphysiological androgen exposure (for example, through anabolic steroid misuse) has been linked to adverse cardiovascular remodeling. Importantly, HRT is increasingly used in men with symptomatic hypogonadism and has demonstrated general CV safety [18,19]. However, evidence suggests that its effects are dose-dependent [20].

**Figure 1:**
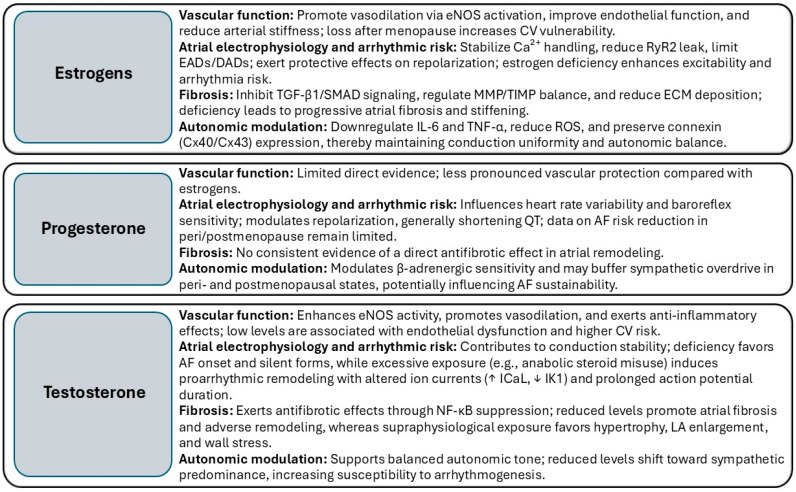
Effects of sex hormones on cardiovascular function, atrial electrophysiology, fibrosis, and autonomic modulation. List of abbreviations: AF: Atrial fibrillation; APD: Action potential duration; Ca^2+^: Calcium ion; CV: Cardiovascular; Cx40/Cx43: Connexin-40/Connexin-43; DADs: Delayed afterdepolarizations; ECM: Extracellular matrix; EADs: Early afterdepolarizations; eNOS: Endothelial nitric oxide synthase; ICaL: L-type calcium current; IK1: Inward rectifier potassium current; IL-6: Interleukin-6; LA: Left atrium; MMP: Matrix metalloproteinase; NF-κB: Nuclear factor kappa-light-chain-enhancer of activated B cells; ROS: Reactive oxygen species; RyR2: Ryanodine receptor type 2; TGF-β1: Transforming growth factor beta 1; TIMP: Tissue inhibitor of metalloproteinases; TNF-α: Tumor necrosis factor alpha; ↑ = increased; ↓ = decreased.

Overall, physiological levels of estrogens and testosterone contribute to the preservation of myocardial structure and function by reducing fibrosis, cardiomyocyte apoptosis, inflammation, and insulin resistance. Both hormones enhance vasodilation, exert antioxidant effects, and promote cellular survival and myocardial perfusion, thereby supporting contractility and maintaining electrophysiological stability. The age-related decline in circulating hormone levels leads to physiological alterations that contribute to sex-specific patterns of HF susceptibility and progression [21]. Fluctuations related to physiological transitions as well as exogenous hormonal therapies, may alter the subtle endocrine-cardiac balance in regulating cardiac structure and function, ultimately influencing the susceptibility to atrial dysfunction and the evolution of arrhythmic phenotypes (Figure 1).

## 3. Hormonal Modulation of Atrial Substrate Remodeling and Atrial Fibrillation Onset

AF arises primarily from ectopic activity and re-entry. Ectopic activity stems from early afterdepolarizations (EADs), caused by prolonged action potential duration via reduced K^+^ currents or increased Na^+^/Ca^2+^ currents, and delayed afterdepolarizations (DADs), driven by sarcoplasmic reticulum Ca^2+^ overload from phospholamban hyperphosphorylation and ryanodine receptor (RyR2) dysfunction. Re-entry can be anatomical or functional, following leading circle or spiral wave patterns, with micro–re-entrant circuits mimicking focal triggers. Myofibroblast–cardiomyocyte coupling through connexin-43 (Cx43) gap junctions facilitates DADs and ectopy via electrotonic interactions. Age-related atrial fibrosis, compounded by autonomic imbalance, further enhances substrate vulnerability for re-entrant arrhythmogenesis [22].

Sex hormones exert direct and multifaceted effects on the molecular architecture of atrial tissue, actively shaping the structural and electrophysiological substrate involved in AF onset [5,23]. Estrogens, and particularly E2, exert electrophysiological stabilizing effects, contributing to the maintenance of a non-arrhythmogenic substrate. At the electrophysiological level, sex differences in calcium handling have emerged as key contributors to atrial vulnerability in AF. In silico human atrial models incorporating sex-specific and AF-associated alterations demonstrated that female atrial cardiomyocytes exhibit a higher incidence of spontaneous calcium release (SCR) events compared to male counterparts, particularly under pacing conditions. This increased arrhythmogenicity is driven primarily by enhanced phosphorylation of RyR2, which promotes diastolic calcium leak and facilitates DADs [24]. Furthermore, ER-β activation within atrial tissue downregulates the expression of pro-inflammatory cytokines such as interleukin-6 (IL-6) and tumor necrosis factor alpha (TNF-α), while suppressing oxidative stress by inhibiting Nicotinamide Adenine Dinucleotide Phosphate (NADPH) oxidase–derived reactive oxygen species (ROS). These effects collectively reduce inflammation-driven electrical remodeling, recognized as a contributor to AF pathogenesis [25,26].

Progesterone, although less extensively studied than estrogens and testosterone, exerts relevant modulatory effects on cardiac electrophysiology and autonomic balance. Beyond its reproductive functions, progesterone receptors are expressed in atrial tissue, where they influence β-adrenergic signaling sensitivity and calcium handling. During the menstrual cycle, fluctuations in progesterone contribute, together with estrogen, to transient changes in heart rate variability and autonomic tone, highlighting its role in the dynamic regulation of atrial excitability [27,28].

In peri- and postmenopausal women, autonomic changes (including reduced heart-rate variability) have been described and may contribute to arrhythmic vulnerability [29,30]. While progesterone has been linked to modulation of repolarization, generally shortening QT in experimental and clinical contexts [31], direct evidence that progesterone per se reduces AF risk in peri-/postmenopause remains limited [32].

Testosterone, under physiological conditions, enhances endothelial NO synthase (eNOS) activity and suppresses the nuclear factor kappa-light-chain-enhancer of activated B cells (NF-κB) pathway, resulting in anti-inflammatory, antifibrotic, and vasoprotective effects. These actions contribute to the maintenance of atrial structural integrity, balanced autonomic tone, and stable conduction properties [17,33]. Conversely, testosterone deficiency is associated with increased atrial fibrosis, impaired connexin-mediated gap junctional communication, and heightened autonomic imbalance, all of which promote conduction heterogeneity and re-entry circuits that facilitate AF initiation and maintenance [5,34,35,36]. At the opposite end of the spectrum, supraphysiological androgen exposure, such as in anabolic steroid abuse, has been linked to proarrhythmic remodeling characterized by upregulation of depolarizing L-type calcium currents (ICaL), downregulation of stabilizing inward rectifier potassium currents (IK1), and elevated oxidative stress. These alterations contribute to action potential prolongation, EADs and DADs, and structural disarray of atrial myocardium, collectively creating a substrate favorable to arrhythmogenesis [37,38,39].

## 4. Remodeling Mechanisms Underlying the Progression of Atrial Fibrillation

The progression from paroxysmal to persistent AF results from a complex interplay of structural, electrical, and inflammatory processes. Key mechanisms, including atrial fibrosis, inflammation, oxidative stress, and autonomic dysregulation, advance independently yet mutually reinforce each other, creating a self-perpetuating pathological loop [40]. Fibrotic remodeling increases conduction heterogeneity and slows electrical propagation, while sustained inflammation and oxidative stress further destabilize ion channel function and enhance ectopic activity. AF maintenance is strongly linked to fibrosis, which disrupts conduction pathways and facilitates re-entry. This process arises from alterations in myocardial fiber architecture, impaired gap junction coupling, and remodeling driven by atrial stretch or tachycardia. Rapid atrial rates stimulate fibroblast-to-myofibroblast differentiation via autocrine/paracrine signaling and Transforming Growth Factor (TGF) β-dependent profibrotic cascades; such effects are attenuated by angiotensin II type 1 receptor blockade [41]. Importantly, sex hormones modulate these core mechanisms (Table 1), acting as endocrine regulators that influence the rate and extent of progression toward persistent AF [42,43].

**Table 1:** Hormonal modulation of atrial substrate remodeling and AF progression.

Arrhythmogenic Mechanisms	Hormonal Modulation	Hormone Deficiency Effects	Excess/Supraphysiological Exposure
Triggered Activity (EADs/DADs)	E2: stabilizes Ca^2+^ handling, ↓ RyR2 phosphorylation, ↓ SCR events; Progesterone: buffers β-adrenergic Ca^2+^ loading; Testosterone: maintains eNOS activity, suppresses NF-κB	↑ Ca^2+^ leak, ↑ SCR events, more DADs, heightened adrenergic sensitivity	Testosterone excess: ↑ ICaL, ↓ IK1, ↑ β-adrenergic responsiveness, ↑ EADs/DADs
Re-entry Substrate	E2: preserves Cx40/Cx43 expression, distribution, phosphorylation; Testosterone: maintains gap junction organization	Disrupted connexin localization, conduction slowing, anisotropy → re-entry facilitation	Testosterone excess: LA enlargement, prolonged conduction delays
Fibrosis/Structural Remodeling	E2: inhibits TGF-β1/SMAD axis, regulates MMP/TIMP balance, ↓ ECM deposition; Testosterone: antifibrotic via NF-κB suppression	↑ TGF-β activity, ↑ collagen synthesis, ECM accumulation, stiffening of atrial wall	Testosterone excess: hypertrophy, wall stress, fibrosis
Inflammation/Oxidative Stress	E2: ER-β–mediated ↓ IL-6/TNF-α, inhibits NADPH oxidase–derived ROS; Testosterone: anti-inflammatory via NF-κB inhibition	↑ pro-inflammatory cytokines, ↑ ROS ↑ electrical instability	Testosterone excess: ↑ oxidative stress, pro-inflammatory signaling
Autonomic Modulation	Progesterone: modulates β-adrenergic sensitivity; Testosterone/E2: maintain autonomic balance	↑ sympathetic tone, ↑ arrhythmia triggers	Testosterone excess: ↑ β-adrenergic responsiveness, vagal effects are context-dependent

List of abbreviations. AF: Atrial fibrillation; EADs: Early afterdepolarizations; DAD: Delayed afterdepolarizations; E2: Estradiol; Ca^2+^: Calcium ion; RyR2: Ryanodine receptor type 2; SCR: Spontaneous calcium release; eNOS: Endothelial nitric oxide synthase; NF-κB: Nuclear factor kappa-light-chain-enhancer of activated B cells; ICaL: L-type calcium current; IK1: Inward rectifier potassium current; Cx40/Cx43: Connexin-40/Connexin-43; ECM: Extracellular matrix; TGF-β: Transforming growth factor beta; MMP: Matrix metalloproteinase; TIMP: Tissue inhibitor of metalloproteinases; IL-6: Interleukin-6; TNF-α—Tumor necrosis factor alpha; NADPH: Nicotinamide Adenine Dinucleotide Phosphate; ROS: Reactive oxygen species; ER-β: Estrogen receptor beta; ↑ = increased; ↓ = decreased; → = results in. References: sex-hormone effects in atrial remodeling, refs. [24,25,26,31,32,33,34,35,36,37,38,39,44,45,46,47,48,49,50,51,52,53,54,55]. Estrogen-related pathways adapted from refs. [24,25,26,44,45,46,47,48,49,50,51,52,53]; testosterone-related evidence from refs. [33,34,35,36,37,38,39,54,55,56]; progesterone-related mechanisms from refs. [27,28,31].

Beyond its acute electrophysiological actions, E2 attenuates atrial fibrosis by downregulating the TGF-β1/SMAD signaling axis, a central driver of fibroblast-to-myofibroblast differentiation and ECM deposition. By limiting the activation of this profibrotic cascade, E2 reduces collagen synthesis and inhibits the structural remodeling that promotes conduction heterogeneity and the formation of fibrotic barriers, which are well-known facilitators of re-entrant electrical activity [44,45,46]. In parallel, E2 regulates the expression and activity of matrix metalloproteinases (MMPs), particularly MMP-2 and MMP-9, and their endogenous inhibitors (TIMPs), thus maintaining ECM turnover in equilibrium. Disruption of this balance, as occurs with estrogen deficiency, leads to progressive matrix accumulation and fibrotic stiffening of the atrial myocardium, enhancing susceptibility to arrhythmogenesis [47,48,49,50].

Furthermore, estrogens affect the long-term rhythm control by modulating the expression and phosphorylation of gap junction proteins, notably Cx40 and Cx43. These connexins are critical for ensuring appropriate atrial conduction velocity and for preventing the formation of re-entrant circuits [51]. Estrogenic signaling regulates the levels, distribution within the plasma membrane, and phosphorylation status of connexins, supporting the preservation of gap junctional communication and conduction uniformity [52]. Long-term estrogen deficiency has been associated with altered connexin localization, impaired intercellular coupling, reduced conduction velocity, and anisotropic propagation, key contributors to wavebreak formation and arrhythmia maintenance [53].

Testosterone modulates atrial remodeling through multifactorial mechanisms that influence the transition from paroxysmal to sustained patterns of AF. At physiological concentrations, androgen signaling helps preserve conduction stability, but experimental evidence indicates a context-dependent role. For example, testosterone replacement in aged rabbit models enhanced arrhythmogenic activity in the pulmonary veins and LA by increasing β-adrenergic responsiveness and upregulating Cav1.2 expression, promoting early and delayed afterdepolarizations (EADs and DADs). Interestingly, despite these proarrhythmic changes, hormone replacement reduced AF inducibility under vagal stimulation, underscoring its complex and nuanced influence on atrial electrophysiology [54]. In states of testosterone depletion, atrial myocytes exhibit lateralization of connexins Cx40 and Cx43, disrupting gap junctional organization and slowing conduction velocity. This disarray contributes to conduction anisotropy and electrical uncoupling, key hallmarks of the arrhythmogenic substrate in persistent AF. Such findings support the clinical observation that hypogonadism is associated with greater AF susceptibility and adverse cardiovascular outcomes [55].

Chronic exposure to supraphysiological doses of anabolic-androgenic steroids, often encountered in athletic contexts, has been associated with pathological cardiac hypertrophy and LA enlargement. These structural changes increase atrial wall stress, promote conduction heterogeneity, and prolong both intra- and inter-atrial electromechanical delay. Together, these effects create a substrate for arrhythmia perpetuation by facilitating inhomogeneous propagation of sinus impulses and re-entrant circuits [56].

## 5. Clinical and Epidemiological Evidence on Hormonal Status in AF Onset and Progression

Sex differences in AF encompass not only prevalence but also age at diagnosis, CV risk profile, symptom burden, and QoL. Epidemiological data consistently show higher AF prevalence in men, who more frequently present with traditional risk factors and structural heart disease [57]. Women, in contrast, are typically diagnosed at an older age and experience a more symptomatic disease course, characterized by greater intensity of palpitations, dyspnea, chest discomfort, and exercise intolerance. These symptoms are associated with significantly lower health-related QoL, particularly in physical functioning domains [58]. Despite exhibiting a lower overall AF burden and shorter episode duration, women demonstrate faster ventricular rates during AF, as shown in large mobile cardiac telemetry datasets [59]. Conversely, men are more likely to present with asymptomatic or silent AF, contributing to delayed diagnoses and increased rates of subclinical episodes. These clinical disparities stem from sex-specific differences in atrial electrophysiology, autonomic regulation, myocardial remodeling, and hormonal influences on cardiac structure and function [60].

In the postmenopausal state, estrogen depletion enhances atrial excitability and imbalances the electrophysiological substrate. The consequent autonomic lability amplifies symptom perception and reduces the detection threshold for AF. Moreover, estrogen level reduction is associated with a prothrombotic state, endothelial dysfunction, and impaired NO bioavailability, which contribute to the disproportionately elevated risk of ischemic stroke and thromboembolism observed in women with AF, even after adjustment for clinical risk factors [61]. However, while sex-related disparities in thromboembolic outcomes remain evident, a distinct pattern emerges when considering the risk of cognitive decline and dementia. Contemporary cohort analyses indicate that, although women continue to experience a relatively higher burden of ischemic stroke compared to men, the strength of this association has attenuated over time, and gender does not translate into a consistent differential risk of dementia when age and comorbidities are accounted for [62].

Long-term observational studies demonstrate that AF is independently associated with accelerated cognitive decline and increased incidence of dementia, irrespective of ischemic stroke events. Interestingly, when stratified by gender, the trajectory of cognitive deterioration appears broadly comparable between men and women, with only subtle differences in specific cognitive domains [63]. Increased risk was confined to non-anticoagulated patients, while anticoagulation mitigated dementia incidence, suggesting benefits extending beyond stroke prevention and implicating subclinical embolic mechanisms [64]. In addition, data from Asian and North American populations suggest that female gender is not an independent determinant of dementia in AF, although older women may exhibit a modestly higher risk, possibly mediated by age-related vascular and hormonal factors [65,66]. A similar age-dependent pattern has been observed for ischemic stroke, where younger women do not show increased risk compared with men, while risk increases in advanced age [67,68]. These observations have led to a critical reappraisal of the CHA_2_DS_2_-VASc score, where female sex has traditionally been incorporated as a risk factor. This reconsideration fostered the development of the CHA_2_DS_2_-VA score, designed to attenuate apparent gender-based disparities in thromboembolic risk assessment and provide a more balanced framework for clinical decision-making [69].

## 6. Hormone-Targeted Therapeutic Strategies in Atrial Fibrillation Management

Despite existing guidelines [70] recommend similar therapeutic approaches for both sexes, hormone-targeted strategies and sex-specific differences in the management of AF represent an emerging area of clinical interest with important implications for treatment personalization (Table 2).

Beyond sex-related anatomical and electrophysiological differences, the hormonal environment shapes therapeutic response and outcomes [71]. Postmenopausal estrogen depletion is linked to autonomic imbalance, enhanced fibrosis, and increased electrical instability. These changes contribute to a more symptomatic disease course in women and partially explain the higher failure rates observed with rhythm-control therapies, including pharmacologic cardioversion and class III antiarrhythmic drugs. Women are also more prone to drug-induced proarrhythmia, such as torsade de pointes, likely due to longer baseline QT intervals and sex-related differences in ion channel expression and drug metabolism [40,72,73].

On the other hand, the age-related decline in circulating testosterone levels in men is closely associated with progressive LA dilation, diffuse interstitial fibrotic remodeling, and maladaptive alterations in ion channel expression and distribution [74,75]. This progression may support a higher efficacy of early rhythm-control strategies, such as electrical cardioversion and ablation, which are more frequently used in men, but also underscores the risk of transitioning from paroxysmal to persistent AF if timely intervention is delayed [76]. Interestingly, testosterone appears to modulate calcium handling and sympathetic responsiveness, and its decline may reduce arrhythmia threshold perception, explaining the higher prevalence of silent or oligosymptomatic AF in aging men, while women experience higher recurrence rates after successful direct current cardioversion [77].

The use of rate-control strategies shows sex- and hormone-related variability. Women, especially in postmenopausal stages, are more frequently treated with digoxin rather than beta-blockers, despite the known association of digoxin with increased all-cause and CV mortality in female patients. This prescribing pattern, observed across several large registries, may reflect sex-specific tolerability issues or a suboptimal adaptation of guideline-based algorithms to female physiology. Furthermore, women undergoing rate-control therapy are more likely to require atrioventricular nodal ablation and pacemaker implantation over time, suggesting a less favorable response to conservative pharmacologic measures [78].

Catheter ablation (CA) of AF has traditionally relied on radiofrequency (RF) energy, with cryoballoon ablation (cryo) increasingly employed as an alternative, and more recently pulsed-field ablation (PFA) emerging as a nonthermal option with promising safety and efficacy profiles [79]. While CA is an increasingly important option for rhythm control, is often underutilized in women, who are referred later and with more advanced atrial remodeling [80]. Estrogen deficiency contributes to a profibrotic substrate, often mediated by upregulation of the TGFβ/Smad3 signaling pathway, which is associated with lower ablation success and higher rates of non-pulmonary vein triggers [81]. Consequently, women are also at increased risk of arrhythmia recurrences after CA, particularly in those with persistent AF [82,83]. Evidence indicates that, while outcomes do not differ by sex in paroxysmal forms, female sex emerges as an independent predictor of recurrence in persistent AF, even after multiple procedures [84]. Furthermore, registry data show that women with non-paroxysmal AF are more frequently treated with additional linear lesions compared with men, underscoring how treatment strategies are often adapted differently by sex and supporting the need for sex-specific approaches in ablation management [85].

Women are also at increased risk of vascular complications and periprocedural adverse events, possibly due to smaller vascular calibers and altered haemostatic profiles, which may be further modulated by hormone status [86]. Women with AF report more symptoms and poorer QoL than men, both before and after CA. Although ablation improves outcomes in both genders, evidence shows that post-procedural symptom burden remains higher in females, and the sex-related QoL is not fully resolved [87,88,89]. Nevertheless, comparative evaluations of these modalities have not identified sex-specific disparities in efficacy [90]. While procedural risk appears consistently elevated among women, the absolute incidence of major complications remains low across both sexes [91]. In contrast, in the MANIFEST-PF registry, PFA demonstrated comparable efficacy and safety between sexes. Despite baseline differences in age, comorbidities, and AF type, no significant sex-related disparities were observed in arrhythmia recurrence or major adverse events at one-year follow-up [92].

With respect to stroke prevention, sex differences are particularly relevant. While warfarin has shown lower protective efficacy in women, partly due to reduced time in therapeutic range, direct oral anticoagulants (DOACs) appear to attenuate this disparity, offering comparable stroke prevention in both genders. However, potential differences in drug metabolism, renal clearance, and bleeding profiles still require further investigation, particularly in women receiving HRT or experiencing abrupt hormonal transitions [93,94].

Treatment disparities may contribute to adverse outcomes to a similar extent as biological differences [95,96]. Nevertheless, when appropriately treated, clinical outcomes are largely comparable between sexes for DOACs, early rhythm control, surgical ablation, and, with the advent of newer technologies, catheter ablation [97,98] (Table 3).

The potential role of HRT, both estrogenic and androgenic, as a preventive or modulatory strategy in AF is gaining attention. Preliminary data suggest that restoring hormonal balance may reduce atrial vulnerability to arrhythmia in selected populations. Nonetheless, manipulating hormone-dependent pathways carries non-negligible risks, including proarrhythmic effects linked to altered ventricular repolarization, QT interval prolongation, and increased susceptibility to thromboembolic or ischemic events. Therefore, the decision to pursue hormone-based interventions in patients with AF must be guided by a rigorous benefit–risk assessment and tailored to the individual’s CV profile and arrhythmic substrate [99,100].

**Table 2:** Sex- and hormone-specific considerations in AF management.

Treatment Strategy	Women (Postmenopause/Estrogen Deficiency)	Men (Gradual Testosterone Decline)	General Considerations
Rhythm control	Greater symptom burden, ↓ success with class III drugs, ↑ risk of TdP; ablation is less used and performed later	Higher efficacy when initiated early; risk of progression to persistent AF if delayed	Personalize strategy according to atrial remodeling and hormonal status
Rate control	More frequent digoxin use (linked to ↑ mortality); higher need for AV nodal ablation/pacemaker	More stable response to β-blockers	Revise algorithms to adapt to sex-specific physiology
Ablation	Lower success due to TGFβ -dependent fibrotic substrate; ↑ vascular complication risk	Higher utilization, often in early stages	Mapping beyond PV may be considered in postmenopausal women
Stroke prevention	Higher thromboembolic risk even at equivalent CHA_2_DS_2_-VA score; DOACs reduce disparity	Risk is more correlated with comorbidities	Monitor drug metabolism and hormonal changes
HRT	Potential to reduce atrial vulnerability; risk of proarrhythmia	Testosterone replacement may improve substrate, but with variable effects on arrhythmogenicity	Selective use after whole risk–benefit evaluation

List of abbreviations. TdP: torsades de points; AF: atrial fibrillation; AV: atrioventricular; TGFβ: transforming growth factor β; PV: pulmonary veins; CHA_2_DS_2_-VA: Congestive heart failure, Hypertension, Age ≥75 (with a double score), Diabetes, Stroke (with a double score), Vascular disease, Age 65–74; DOACs: direct oral anticoagulations; HRT: hormone replacement therapy; ↑ = increased; ↓ = decreased. References: Rhythm/rate control: refs. [70,71,72,73,76,77,78]; ablation efficacy and complications (incl. PFA): refs. [79,80,81,82,83,84,85,86,87,88,89,90,91,92]; stroke prevention (warfarin/DOACs): refs. [93,94,95]; hormonal therapy considerations: refs. [13,18,19,20,100,101,102,103].

**Table 3:** Treatment outcomes between women and men in atrial fibrillation.

Treatment	Efficacy	Safety
Anticoagulation (DOAC vs. VKA)	Comparable benefit in both sexes: ↓ stroke/SE	DOAC: ↓ major bleeding and ICH vs. VKA in both sexes
Catheter ablation (RF and Cryo techniques)	Slightly higher recurrence in women, especially in persistent AF; others report no major differences.	Slightly higher periprocedural complications in women (vascular/bleeding) no significant differences.
Catheter ablation—Pulsed Field Ablation (PFA)	No significant sex differences in 1-year freedom from AF/AT recurrence.	Similar safety profile overall; one analysis reported slightly higher acute complications in women, but absolute rates were low.
Surgical (Cox-Maze/surgical ablation)	Long-term outcomes are similar between sexes (SR maintenance, survival, QoL).	Comparable safety profile across sexes in historical and adjusted cohorts.

List of abbreviations: AF: atrial fibrillation; AT: atrial tachycardia; Cryo: cryoablation; DOAC: direct oral anticoagulant; ICH: intracranial hemorrhage; QoL: quality of life; RF: radiofrequency; SE: systemic embolism; SR: sinus rhythm; VKA: vitamin K antagonist; ↓ = decreased. References: Anticoagulation (DOAC vs. VKA): refs. [93,94,95]. Catheter ablation (RF/cryo): efficacy/recurrence and strategy: refs. [80,82,83,84,85,90,91]; safety/complications and patient-reported outcomes: refs. [81,86,87,88,89]. Catheter ablation (PFA): refs. [79,92]. Surgical ablation (Cox-Maze): refs. [98].

Patient-specific approaches are essential: estrogen-containing therapy may be considered in carefully selected postmenopausal women with symptomatic AF and low thromboembolic risk [101] while testosterone replacement in men with clinically relevant hypogonadism should target physiological ranges [102]; conversely, caution is warranted with estrogen therapy in patients at high venous thromboembolism risk [103] and with androgen therapy in settings prone to erythrocytosis or prothrombotic states [17].

Decision-making algorithms for AF may be strengthened by including hormonal status as a complementary parameter to traditional clinical predictors. Patient profiling (sex, menopausal status, hypogonadism, comorbidities), evaluation of AF burden, atrial remodeling, and standard risk scores (CHA_2_DS_2_-VA, bleeding and proarrhythmic risk) can be integrated to refine rhythm- or rate-control strategies, guide timing of ablation, and optimize anticoagulation. In this way, hormonal status contributes not as an isolated factor but as part of a broader, individualized approach.

## 7. Future Perspectives and Conclusions

Advances in the understanding of sex-specific determinants of AF are paving the way toward precision medicine approaches that integrate hormonal status, structural substrate, and comorbidity profile into therapeutic decision-making. Clinically, the recognition that repolarizing ion currents are lower and action potential duration is prolonged in female atria has relevant implications for rhythm-control strategies. Class III antiarrhythmics may achieve atrial antiarrhythmic efficacy at lower dosages in women, but their use necessitates strict surveillance due to an inherently greater susceptibility to QT prolongation and ventricular pro-arrhythmia. The higher prevalence of non-pulmonary vein triggers in women suggests that ablation strategies should incorporate systematic mapping beyond the pulmonary veins to enhance procedural success rates, particularly in postmenopausal patients with advanced substrate remodeling [75]. In persistent AF, women exhibit more extensive low-voltage atrial substrate despite comparable chamber size, with earlier and regionally accentuated remodeling; voltage-guided ablation appears to mitigate outcome differences [104]. Complementarily, artificial intelligence (AI) applied to cardiology offers substantial advantages, including improved efficiency and accuracy in electrocardiogram (ECG) interpretation, and in this context an AI-based ECG sex-discrepancy marker identifies women with greater atrial enlargement and higher post-ablation recurrence risk, suggesting sex-specific substrate characterization can refine prognosis and procedural planning [105,106]. Over the next years, AI may support a more individualized management of AF, guiding therapeutic decisions across anticoagulation, rhythm- versus rate-control, and ablation strategies [107].

Given the more pronounced atrial fibrotic remodeling observed in women, often linked to TGFβ/Smad3 pathway activation, this group could gain even greater benefit from antifibrotic therapies, such as angiotensin receptor blockers, mineralocorticoid receptor antagonists, and emerging pathway-specific inhibitors. For this reason, their use should be encouraged and given higher priority in this population [108,109,110,111]. In parallel, the heightened pro-inflammatory milieu associated with epicardial fat distribution in older women identifies anti-inflammatory interventions, pharmacological or lifestyle-based, as promising adjuncts.

Epicardial adipose tissue (EAT) has been implicated in the pathogenesis of the obesity–AF relation in both genders. A local paracrine effect of EAT mediated by inflammatory cytokines, growth and remodeling factors, angiogenic factors, and adipocytokines may lead to the development of the AF substrate. EAT location on CT imaging correlates with high dominant excitation frequency during electroanatomic mapping in patients undergoing AF ablation [112,113].

The higher prevalence of HF with preserved ejection fraction (HFpEF) and microvascular dysfunction underscores the importance of addressing these comorbidities with targeted agents to improve atrial substrate stability [75].

Emerging pharmacological approaches with demonstrated CV benefit, such as sodium–glucose cotransporter 2 (SGLT2) inhibitors and glucagon-like peptide-1 receptor agonists (GLP-1RAs), exert multifactorial antifibrotic, anti-inflammatory, and metabolic effects, which may be particularly advantageous in hormone-deficient states such as post menopause or late-onset hypogonadism, where these pathological processes are amplified [112,114].

Additional investigational strategies target key molecular determinants of atrial structural remodeling and fibrosis, also influenced by sex hormone signaling. For example, microRNA-based therapeutics, such as the upregulation of miR-29 or miR-133 to suppress collagen synthesis [41], may counteract the profibrotic gene expression profile observed with estrogen depletion, while epigenetic modulators, including histone deacetylase inhibitors and methyltransferase inhibitors such as EZH2 antagonists, could reverse chromatin changes linked to androgen or estrogen deficiency [110]. Furthermore, Cx43, the principal atrial gap junction protein, is regulated in part through estrogen receptor-dependent pathways; therapeutic strategies that preserve or restore its expression and distribution may reduce sex-specific differences in conduction heterogeneity and susceptibility to arrhythmia [115].

Despite significant advances, key uncertainties persist regarding the relative impact of estrogen loss, androgen decline, and comorbidity-driven remodeling on sex-specific AF progression. These mechanisms are often interdependent, and their individual contribution remains incompletely defined. The persistent underrepresentation of women in randomized trials further limits the development of robust, sex-specific recommendations, perpetuating treatment paradigms that may inadequately reflect biological and clinical differences.

Future research should prioritize mechanistic studies in sex-stratified human atrial tissue, incorporation of hormonal profiling into AF risk assessment, and prospective trials evaluating tailored pharmacological regimens, ablation strategies, and adjunctive antifibrotic or anti-inflammatory therapies in hormone-deficient states. Such an approach could align therapeutic decisions with the patient’s hormonal status, structural remodeling patterns, and comorbidity profile, ultimately improving efficacy and safety.

Integrating these insights into clinical algorithms is essential to advance beyond a “one-size-fits-all” model toward truly individualized AF management. Precision strategies that recognize and address sex-specific determinants of arrhythmia could redefine current standards of care maximizing benefit while minimizing harm. This shift is a necessary step toward equitable, evidence-based, and outcome-driven cardiology.

## Data Availability

Data are contained within the article.

## Abbreviations

The following abbreviations are used in this manuscript:

AF	Atrial fibrillation
AI	Artificial intelligence
AR	Androgen receptor
CA	Catheter ablation
Cryo	Cryoballoon
CHA_2_DS_2_-VA	Congestive heart failure–Hypertension–Age–Diabetes–Stroke/Thromboembolism–Vascular disease–Age (65–74) score (without Sex)
CHA_2_DS_2_-VASc	Congestive heart failure–Hypertension–Age–Diabetes–Stroke/Thromboembolism–Vascular disease–Age (65–74)–Sex category score
Cx40	Connexin 40
Cx43	Connexin 43
CV	Cardiovascular
DAD	Delayed afterdepolarization
DOAC	Direct oral anticoagulant
E2	Estradiol
EAD	Early afterdepolarization
EAT	Epicardial adipose tissue
ECG	Electrocardiogram
ECM	Extracellular matrix
eNOS	Endothelial nitric oxide synthase
ER-α	Estrogen receptor alpha
ER-β	Estrogen receptor beta
GLP-1RA	Glucagon-like peptide-1 receptor agonist
HF	Heart failure
HFpEF	Heart failure with preserved ejection fraction
HRT	Hormone replacement therapy
ICaL	L-type calcium current
IK1	Inward rectifier potassium current
IL-6	Interleukin-6
LA	Left atrium/left atrial
LVZ	Low-voltage zone
MANIFEST-PF	Multinational Survey on the Methods, Efficacy, and Safety on the Postapproval Clinical Use of Pulsed Field Ablation
MMP	Matrix metalloproteinase
MRI	Magnetic resonance imaging
NADPH	Nicotinamide adenine dinucleotide phosphate
NF-κB	Nuclear factor kappa-light-chain-enhancer of activated B cells
NO	Nitric oxide
PFA	Pulsed-field ablation
PVs	Pulmonary veins
QoL	Quality of life
RF	Radiofrequency
ROS	Reactive oxygen species
RyR2	Ryanodine receptor 2
SCR	Spontaneous calcium release
SGLT2	Sodium–glucose cotransporter 2
Smad3	Mothers against decapentaplegic homolog 3
TGF-β	Transforming growth factor beta
TIMPs	Tissue inhibitors of metalloproteinases
TNF-α	Tumor necrosis factor alpha

## References

[1] Schnabel R.B., Yin X., Gona P., Larson M.G., Beiser A.S., McManus D.D., Newton-Cheh C., Lubitz S.A., Magnani J.W., Ellinor P.T. (2015). 50 Year Trends in Atrial Fibrillation Prevalence, Incidence, Risk Factors, and Mortality in the Framingham Heart Study: A Cohort Study. Lancet.

[2] Peters R.W., Gold M.R. (2004). The Influence of Gender on Arrhythmias. Cardiol. Rev..

[3] Westerman S., Wenger N. (2019). Gender Differences in Atrial Fibrillation: A Review of Epidemiology, Management, and Outcomes. CCR.

[4] Burgess S., Zaman S., Towns C., Coylewright M., Cader F.A. (2025). The Under-Representation of Women in Cardiovascular Clinical Trials: State-of-the-Art Review and Ethical Considerations. Am. Heart J..

[5] Magnani J.W., Moser C.B., Murabito J.M., Sullivan L.M., Wang N., Ellinor P.T., Vasan R.S., Benjamin E.J., Coviello A.D. (2014). Association of Sex Hormones, Aging, and Atrial Fibrillation in Men: The Framingham Heart Study. Circ. Arrhythmia Electrophysiol..

[6] Menazza S., Murphy E. (2016). The Expanding Complexity of Estrogen Receptor Signaling in the Cardiovascular System. Circ. Res..

[7] Walker C.J., Schroeder M.E., Aguado B.A., Anseth K.S., Leinwand L.A. (2021). Matters of the Heart: Cellular Sex Differences. J. Mol. Cell. Cardiol..

[8] Bukovsky A., Caudle M.R., Cekanova M., Fernando R.I., Wimalasena J., Foster J.S., Henley D.C., Elder R.F. (2003). Placental Expression of Estrogen Receptor Beta and Its Hormone Binding Variant–Comparison with Estrogen Receptor Alpha and a Role for Estrogen Receptors in Asymmetric Division and Differentiation of Estrogen-Dependent Cells. Reprod. Biol. Endocrinol..

[9] Murphy E. (2011). Estrogen Signaling and Cardiovascular Disease. Circ. Res..

[10] Minson C.T., Halliwill J.R., Young T.M., Joyner M.J. (2000). Influence of the Menstrual Cycle on Sympathetic Activity, Baroreflex Sensitivity, and Vascular Transduction in Young Women. Circulation.

[11] Ryczkowska K., Adach W., Janikowski K., Banach M., Bielecka-Dabrowa A. (2023). Menopause and Women’s Cardiovascular Health: Is It Really an Obvious Relationship?. Arch. Med. Sci..

[12] Maas A.H.E.M., Rosano G., Cifkova R., Chieffo A., Van Dijken D., Hamoda H., Kunadian V., Laan E., Lambrinoudaki I., Maclaran K. (2021). Cardiovascular Health after Menopause Transition, Pregnancy Disorders, and Other Gynaecologic Conditions: A Consensus Document from European Cardiologists, Gynaecologists, and Endocrinologists. Eur. Heart J..

[13] Cho L., Kaunitz A.M., Faubion S.S., Hayes S.N., Lau E.S., Pristera N., Scott N., Shifren J.L., Shufelt C.L., Stuenkel C.A. (2023). Rethinking Menopausal Hormone Therapy: For Whom, What, When, and How Long?. Circulation.

[14] El Khoudary S.R., Aggarwal B., Beckie T.M., Hodis H.N., Johnson A.E., Langer R.D., Limacher M.C., Manson J.E., Stefanick M.L., Allison M.A. (2020). Menopause Transition and Cardiovascular Disease Risk: Implications for Timing of Early Prevention: A Scientific Statement from the American Heart Association. Circulation.

[15] Schafstedde M., Nordmeyer S. (2023). The Role of Androgens in Pressure Overload Myocardial Hypertrophy. Front. Endocrinol..

[16] Babcock M.C., DuBose L.E., Witten T.L., Stauffer B.L., Hildreth K.L., Schwartz R.S., Kohrt W.M., Moreau K.L. (2022). Oxidative Stress and Inflammation Are Associated with Age-Related Endothelial Dysfunction in Men with Low Testosterone. J. Clin. Endocrinol. Metab..

[17] Oskui P.M., French W.J., Herring M.J., Mayeda G.S., Burstein S., Kloner R.A. (2013). Testosterone and the Cardiovascular System: A Comprehensive Review of the Clinical Literature. JAHA.

[18] Lincoff A.M., Bhasin S., Flevaris P., Mitchell L.M., Basaria S., Boden W.E., Cunningham G.R., Granger C.B., Khera M., Thompson I.M. (2023). Cardiovascular Safety of Testosterone-Replacement Therapy. N. Engl. J. Med..

[19] Jaiswal V., Sawhney A., Nebuwa C., Borra V., Deb N., Halder A., Rajak K., Jha M., Wajid Z., Thachil R. (2024). Association between Testosterone Replacement Therapy and Cardiovascular Outcomes: A Meta-Analysis of 30 Randomized Controlled Trials. Prog. Cardiovasc. Dis..

[20] Mason F.E., Liutkute A., Voigt N. (2025). Testosterone and Atrial Fibrillation: Does the Dose Make the Poison?. Cardiovasc. Res..

[21] Ebong I.A., Appiah D., Mauricio R., Narang N., Honigberg M.C., Ilonze O.J., Aggarwal N.R., Zanni M.V., Mohammed S.F., Cho L. (2025). Sex Hormones and Heart Failure Risk. JACC Adv..

[22] Curcio A., Torella D., Iaconetti C., Pasceri E., Sabatino J., Sorrentino S., Giampà S., Micieli M., Polimeni A., Henning B.J. (2013). MicroRNA-1 Downregulation Increases Connexin 43 Displacement and Induces Ventricular Tachyarrhythmias in Rodent Hypertrophic Hearts. PLoS ONE.

[23] Ko D., Rahman F., Schnabel R.B., Yin X., Benjamin E.J., Christophersen I.E. (2016). Atrial Fibrillation in Women: Epidemiology, Pathophysiology, Presentation, and Prognosis. Nat. Rev. Cardiol..

[24] Zhang X., Wu Y., Smith C.E.R., Louch W.E., Morotti S., Dobrev D., Grandi E., Ni H. (2024). Enhanced Ca2+-Driven Arrhythmogenic Events in Female Patients with Atrial Fibrillation. JACC Clin. Electrophysiol..

[25] Dantas A.P.V., Sandberg K. (2005). Estrogen Regulation of Tumor Necrosis Factor-α: A Missing Link Between Menopause and Cardiovascular Risk in Women?. Hypertension.

[26] Ramos-Mondragón R., Lozhkin A., Vendrov A.E., Runge M.S., Isom L.L., Madamanchi N.R. (2023). NADPH Oxidases and Oxidative Stress in the Pathogenesis of Atrial Fibrillation. Antioxidants.

[27] Yildirir A., Kabakci G., Akgul E., Tokgozoglu L., Oto A. (2001). Effects of Menstrual Cycle on Cardiac Autonomic Innervation As Assessed By Heart Rate Variability. Noninvasive Electrocardiol..

[28] Schmalenberger K.M., Eisenlohr-Moul T.A., Jarczok M.N., Eckstein M., Schneider E., Brenner I.G., Duffy K., Schweizer S., Kiesner J., Thayer J.F. (2020). Menstrual Cycle Changes in Vagally-Mediated Heart Rate Variability Are Associated with Progesterone: Evidence from Two Within-Person Studies. J. Clin. Med..

[29] La Rovere M.T., Pinna G.D., Hohnloser S.H., Marcus F.I., Mortara A., Nohara R., Bigger J.T., Camm A.J., Schwartz P.J. (2001). Baroreflex Sensitivity and Heart Rate Variability in the Identification of Patients at Risk for Life-Threatening Arrhythmias: Implications for Clinical Trials. Circulation.

[30] Souza H.C.D., Tezini G.C.S.V. (2013). Autonomic Cardiovascular Damage during Post-Menopause: The Role of Physical Training. Aging Dis..

[31] Nakamura H., Kurokawa J., Bai C.-X., Asada K., Xu J., Oren R.V., Zhu Z.I., Clancy C.E., Isobe M., Furukawa T. (2007). Progesterone Regulates Cardiac Repolarization Through a Nongenomic Pathway: An In Vitro Patch-Clamp and Computational Modeling Study. Circulation.

[32] Wong J.A., Rexrode K.M., Sandhu R.K., Moorthy M.V., Conen D., Albert C.M. (2017). Menopausal Age, Postmenopausal Hormone Therapy and Incident Atrial Fibrillation. Heart.

[33] Jin H., Qiu W.-B., Mei Y.-F., Wang D.-M., Li Y.-G., Tan X.-R. (2009). Testosterone Alleviates Tumor Necrosis Factor-Alpha-Mediated Tissue Factor Pathway Inhibitor Downregulation via Suppression of Nuclear Factor-Kappa B in Endothelial Cells. Asian J. Androl..

[34] Tsuneda T., Yamashita T., Kato T., Sekiguchi A., Sagara K., Sawada H., Aizawa T., Fu L., Fujiki A., Inoue H. (2009). Deficiency of Testosterone Associates with the Substrate of Atrial Fibrillation in the Rat Model. Cardiovasc. Electrophysiol..

[35] Zeller T., Schnabel R.B., Appelbaum S., Ojeda F., Berisha F., Schulte-Steinberg B., Brueckmann B.-E., Kuulasmaa K., Jousilahti P., Blankenberg S. (2018). Low Testosterone Levels Are Predictive for Incident Atrial Fibrillation and Ischaemic Stroke in Men, but Protective in Women–Results from the FINRISK Study. Eur. J. Prev. Cardiolog..

[36] Andelova K., Egan Benova T., Szeiffova Bacova B., Sykora M., Prado N.J., Diez E.R., Hlivak P., Tribulova N. (2020). Cardiac Connexin-43 Hemichannels and Pannexin1 Channels: Provocative Antiarrhythmic Targets. Int. J. Mol. Sci..

[37] Tran C., Yeap B.B., Ball J., Clayton-Chubb D., Hussain S.M., Brodtmann A., Tonkin A.M., Neumann J.T., Schneider H.G., Fitzgerald S. (2024). Testosterone and the Risk of Incident Atrial Fibrillation in Older Men: Further Analysis of the ASPREE Study. eClinicalMedicine.

[38] Sharma R., Oni O.A., Gupta K., Sharma M., Sharma R., Singh V., Parashara D., Kamalakar S., Dawn B., Chen G. (2017). Normalization of Testosterone Levels After Testosterone Replacement Therapy Is Associated with Decreased Incidence of Atrial Fibrillation. JAHA.

[39] Zhang Y., Wang H.-M., Wang Y.-Z., Zhang Y.-Y., Jin X.-X., Zhao Y., Wang J., Sun Y.-L., Xue G.-L., Li P.-H. (2017). Increment of Late Sodium Currents in the Left Atrial Myocytes and Its Potential Contribution to Increased Susceptibility of Atrial Fibrillation in Castrated Male Mice. Heart Rhythm..

[40] Curcio A., Scalise R., Indolfi C. (2024). Pathophysiology of Atrial Fibrillation and Approach to Therapy in Subjects Less than 60 Years Old. Int. J. Mol. Sci..

[41] Heijman J., Guichard J.-B., Dobrev D., Nattel S. (2018). Translational Challenges in Atrial Fibrillation. Circ. Res..

[42] Karakasis P., Theofilis P., Vlachakis P.K., Korantzopoulos P., Patoulias D., Antoniadis A.P., Fragakis N. (2024). Atrial Fibrosis in Atrial Fibrillation: Mechanistic Insights, Diagnostic Challenges, and Emerging Therapeutic Targets. Int. J. Mol. Sci..

[43] Pang Z., Ren Y., Yao Z. (2025). Interactions between Atrial Fibrosis and Inflammation in Atrial Fibrillation. Front. Cardiovasc. Med..

[44] Pedram A., Razandi M., O’Mahony F., Lubahn D., Levin E.R. (2010). Estrogen Receptor-β Prevents Cardiac Fibrosis. Mol. Endocrinol..

[45] Gramley F., Lorenzen J., Koellensperger E., Kettering K., Weiss C., Munzel T. (2010). Atrial Fibrosis and Atrial Fibrillation: The Role of the TGF-Β1 Signaling Pathway. Int. J. Cardiol..

[46] Xu J., Wang F., Li Y., Li P., Zhang Y., Xu G., Sun K. (2024). Estrogen Inhibits TGF-β1-stimulated Cardiac Fibroblast Differentiation and Collagen Synthesis by Promoting Cdc42. Mol. Med. Rep..

[47] Linssen P.B.C., Brunner-La Rocca H.-P., Schalkwijk C.G., Beulens J.W.J., Elders P.J.M., Van Der Heijden A.A., Slieker R.C., Stehouwer C.D.A., Henry R.M.A. (2020). Serum Matrix Metalloproteinases and Left Atrial Remodeling—The Hoorn Study. Int. J. Mol. Sci..

[48] Mahmoodzadeh S., Dworatzek E., Fritschka S., Pham T.H., Regitz-Zagrosek V. (2010). 17β-Estradiol Inhibits Matrix Metalloproteinase-2 Transcription via MAP Kinase in Fibroblasts. Cardiovasc. Res..

[49] Sonmez O., Ertem F.U., Vatankulu M.A., Erdogan E., Tasal A., Kucukbuzcu S., Goktekin O. (2014). Novel Fibro-Inflammation Markers in Assessing Left Atrial Remodeling in Non-Valvular Atrial Fibrillation. Med. Sci. Monit..

[50] Voloshenyuk T.G., Gardner J.D. (2010). Estrogen Improves TIMP-MMP Balance and Collagen Distribution in Volume-Overloaded Hearts of Ovariectomized Females. Am. J. Physiol. Regul. Integr. Comp. Physiol..

[51] Rodríguez-Sinovas A., Sánchez J.A., Valls-Lacalle L., Consegal M., Ferreira-González I. (2021). Connexins in the Heart: Regulation, Function and Involvement in Cardiac Disease. Int. J. Mol. Sci..

[52] Oyamada M., Takebe K., Oyamada Y. (2013). Regulation of Connexin Expression by Transcription Factors and Epigenetic Mechanisms. Biochim. Biophys. Acta (BBA)-Biomembr..

[53] Ai X., Yan J., Pogwizd S.M. (2021). Serine-Threonine Protein Phosphatase Regulation of Cx43 Dephosphorylation in Arrhythmogenic Disorders. Cell. Signal..

[54] Tsai W.-C., Lee T.-I., Chen Y.-C., Kao Y.-H., Lu Y.-Y., Lin Y.-K., Chen S.-A., Chen Y.-J. (2014). Testosterone Replacement Increases Aged Pulmonary Vein and Left Atrium Arrhythmogenesis with Enhanced Adrenergic Activity. Int. J. Cardiol..

[55] Thibault S., Ton A.-T., Huynh F., Fiset C. (2022). Connexin Lateralization Contributes to Male Susceptibility to Atrial Fibrillation. Int. J. Mol. Sci..

[56] Akçakoyun M., Alizade E., Gündoğdu R., Bulut M., Tabakcı M.M., Açar G., Avcı A., Şimşek Z., Fidan S., Demir S. (2014). Long-Term Anabolic Androgenic Steroid Use Is Associated with Increased Atrial Electromechanical Delay in Male Bodybuilders. BioMed Res. Int..

[57] Magnussen C., Ojeda F.M., Wild P.S., Sörensen N., Rostock T., Hoffmann B.A., Prochaska J., Lackner K.J., Beutel M.E., Blettner M. (2018). Atrial Fibrillation Manifestations Risk Factors and Sex Differences in a Population-Based Cohort (From the Gutenberg Health Study). Am. J. Cardiol..

[58] Piccini J.P., Simon D.N., Steinberg B.A., Thomas L., Allen L.A., Fonarow G.C., Gersh B., Hylek E., Kowey P.R., Reiffel J.A. (2016). Differences in Clinical and Functional Outcomes of Atrial Fibrillation in Women and Men: Two-Year Results from the ORBIT-AF Registry. JAMA Cardiol..

[59] Tan J.L., Johnson L., Dziubinski M., Napiorkowski N., Witkowska O., Slusarczyk M.E., Healey J.S., Russo A.M. (2022). Sex Differences in Presentation of Atrial Fibrillation: Findings from 30-Day Ambulatory Monitoring in Real-World Practice. Am. Heart J. Plus Cardiol. Res. Pract..

[60] Blum S., Muff C., Aeschbacher S., Ammann P., Erne P., Moschovitis G., Di Valentino M., Shah D., Schläpfer J., Fischer A. (2017). Prospective Assessment of Sex-Related Differences in Symptom Status and Health Perception Among Patients with Atrial Fibrillation. JAHA.

[61] Kostopoulou A., Zeljko H.M., Bogossian H., Ciudin R., Costa F., Heijman J., Kochhaeuser S., Manola S., Scherr D., Sohal M. (2020). Atrial Fibrillation-related Stroke in Women: Evidence and Inequalities in Epidemiology, Mechanisms, Clinical Presentation, and Management. Clin. Cardiol..

[62] Mills M.T., Bucci T., Calvert P., Gupta D., Lip G.Y.H. (2025). Temporal Trends in the Association Between Female Sex and Ischemic Stroke Among Patients with Atrial Fibrillation. JAHA.

[63] Chen L.Y., Norby F.L., Gottesman R.F., Mosley T.H., Soliman E.Z., Agarwal S.K., Loehr L.R., Folsom A.R., Coresh J., Alonso A. (2018). Association of Atrial Fibrillation with Cognitive Decline and Dementia Over 20 Years: The ARIC-NCS (Atherosclerosis Risk in Communities Neurocognitive Study). JAHA.

[64] Field T.S., Weijs B., Curcio A., Giustozzi M., Sudikas S., Katholing A., Wallenhorst C., Weitz J.I., Cohen A.T., Martinez C. (2019). Incident Atrial Fibrillation, Dementia and the Role of Anticoagulation: A Population-Based Cohort Study. Thromb. Haemost..

[65] Chen Y.-L., Chen J., Wang H.-T., Chang Y.-T., Chong S.-Z., Hsueh S., Chung C.-M., Lin Y.-S. (2021). Sex Difference in the Risk of Dementia in Patients with Atrial Fibrillation. Diagnostics.

[66] Golive A., May H.T., Bair T.L., Jacobs V., Crandall B.G., Cutler M.J., Day J.D., Mallender C., Osborn J.S., Weiss J.P. (2018). The Impact of Gender on Atrial Fibrillation Incidence and Progression to Dementia. Am. J. Cardiol..

[67] Wu V.C.-C., Wu M., Aboyans V., Chang S.-H., Chen S.-W., Chen M.-C., Wang C.-L., Hsieh I.-C., Chu P.-H., Lin Y.-S. (2020). Female Sex as a Risk Factor for Ischaemic Stroke Varies with Age in Patients with Atrial Fibrillation. Heart.

[68] Mikkelsen A.P., Lindhardsen J., Lip G.Y.H., Gislason G.H., Torp-Pedersen C., Olesen J.B. (2012). Female Sex as a Risk Factor for Stroke in Atrial Fibrillation: A Nationwide Cohort Study. J. Thromb. Haemost..

[69] Clua-Espuny J.L., Panisello-Tafalla A., Lucas-Noll J., Muria-Subirats E., Forcadell-Arenas T., Carrera-Ortiz J.M., Molto-Balado P., Clua-Queralt J., Fusté-Anguera I., Reverte-Vilarroya S. (2025). Stroke Risk Stratification in Incident Atrial Fibrillation: A Sex-Specific Evaluation of CHA2DS2-VA and CHA2DS2-VASc. JCDD.

[70] Van Gelder I.C., Rienstra M., Bunting K.V., Casado-Arroyo R., Caso V., Crijns H.J.G.M., De Potter T.J.R., Dwight J., Guasti L., Hanke T. (2024). 2024 ESC Guidelines for the Management of Atrial Fibrillation Developed in Collaboration with the European Association for Cardio-Thoracic Surgery (EACTS). Eur. Heart J..

[71] Lip G.Y.H., Laroche C., Boriani G., Cimaglia P., Dan G.-A., Santini M., Kalarus Z., Rasmussen L.H., Popescu M.I., Tica O. (2015). Sex-Related Differences in Presentation, Treatment, and Outcome of Patients with Atrial Fibrillation in Europe: A Report from the Euro Observational Research Programme Pilot Survey on Atrial Fibrillation. EP Eur..

[72] Rienstra M., Van Veldhuisen D.J., Hagens V.E., Ranchor A.V., Veeger N.J.G.M., Crijns H.J.G.M., Van Gelder I.C. (2005). Gender-Related Differences in Rhythm Control Treatment in Persistent Atrial Fibrillation. J. Am. Coll. Cardiol..

[73] Essebag V. (2007). Sex Differences in the Relationship Between Amiodarone Use and the Need for Permanent Pacing in Patients with Atrial Fibrillation. Arch. Intern. Med..

[74] Nikitin N.P., Witte K.K.A., Thackray S.D.R., Goodge L.J., Clark A.L., Cleland J.G.F. (2003). Effect of Age and Sex on Left Atrial Morphology and Function. Eur. Heart J.-Cardiovasc. Imaging.

[75] Odening K.E., Deiß S., Dilling-Boer D., Didenko M., Eriksson U., Nedios S., Ng F.S., Roca Luque I., Sanchez Borque P., Vernooy K. (2019). Mechanisms of Sex Differences in Atrial Fibrillation: Role of Hormones and Differences in Electrophysiology, Structure, Function, and Remodelling. EP Eur..

[76] Schnabel R.B., Pecen L., Ojeda F.M., Lucerna M., Rzayeva N., Blankenberg S., Darius H., Kotecha D., Caterina R.D., Kirchhof P. (2017). Gender Differences in Clinical Presentation and 1-Year Outcomes in Atrial Fibrillation. Heart.

[77] Gurevitz O.T., Varadachari C.J., Ammash N.M., Malouf J.F., Rosales A.G., Herges R.M., Bruce C.J., Somers V.K., Hammill S.C., Gersh B.J. (2006). The Effect of Patient Sex on Recurrence of Atrial Fibrillation Following Successful Direct Current Cardioversion. Am. Heart J..

[78] Kassim N.A., Althouse A.D., Qin D., Leef G., Saba S. (2017). Gender Differences in Management and Clinical Outcomes of Atrial Fibrillation Patients. J. Cardiol..

[79] Calvert P., Mills M.T., Xydis P., Essa H., Ding W.Y., Koniari I., Farinha J.M., Harding M., Mahida S., Snowdon R. (2024). Cost, Efficiency, and Outcomes of Pulsed Field Ablation vs Thermal Ablation for Atrial Fibrillation: A Real-World Study. Heart Rhythm..

[80] Duarte F., Silva-Teixeira R., Aguiar-Neves I., Almeida J.G., Fonseca P., Monteiro A.V., Oliveira M., Gonçalves H., Ribeiro J., Caramelo F. (2025). Sex Differences in Atrial Remodeling and Atrial Fibrillation Recurrence after Catheter Ablation. Heart Rhythm..

[81] Vandenberk B., Chew D.S., Parkash R., Gillis A.M. (2022). Sex and Racial Disparities in Catheter Ablation. Heart Rhythm O2.

[82] Du W., Zhu W., Yang H., Dong Q., Fei Y., Li X., Li S., Han B. (2025). Different Impact of Female Gender on the Outcome of Catheter Ablation between Paroxysmal and Persistent Atrial Fibrillation. BMC Cardiovasc. Disord..

[83] Al-Sadawi M., Aslam F., Gier C., Aleem S., Ijaz H., Jacobs R., Cao K., Alsaiqali M., Singh A. (2023). Effect of Gender on Atrial Fibrillation Ablation Outcomes Using a Propensity Score–Matched Analysis. Heart Rhythm O2.

[84] Sugumar H., Nanayakkara S., Chieng D., Wong G.R., Parameswaran R., Anderson R.D., Al-Kaisey A., Nalliah C.J., Azzopardi S., Prabhu S. (2020). Arrhythmia Recurrence Is More Common in Females Undergoing Multiple Catheter Ablation Procedures for Persistent Atrial Fibrillation: Time to Close the Gender Gap. Heart Rhythm..

[85] Yunus F.N., Perino A.C., Holmes D.N., Matsouaka R.A., Curtis A.B., Ellenbogen K.A., Frankel D.S., Knight B.P., Russo A.M., Lewis W.R. (2021). Sex Differences in Ablation Strategy, Lesion Sets, and Complications of Catheter Ablation for Atrial Fibrillation: An Analysis from the GWTG-AFIB Registry. Circ. Arrhythmia Electrophysiol..

[86] Kaiser D.W., Fan J., Schmitt S., Than C.T., Ullal A.J., Piccini J.P., Heidenreich P.A., Turakhia M.P. (2016). Gender Differences in Clinical Outcomes After Catheter Ablation of Atrial Fibrillation. JACC Clin. Electrophysiol..

[87] Mills M.T., Lip G.Y.H., Luther V., Gupta D. (2025). Sex-Based Differences in Symptomatology in the First Month Following Atrial Fibrillation Catheter Ablation. Cardiovasc. Electrophysiol..

[88] Zeitler E.P., Li Y., Silverstein A.P., Russo A.M., Poole J.E., Daniels M.R., Al-Khalidi H.R., Lee K.L., Bahnson T.D., Anstrom K.J. (2023). Effects of Ablation Versus Drug Therapy on Quality of Life by Sex in Atrial Fibrillation: Results from the CABANA Trial. JAHA.

[89] Yao R.J.R., Macle L., Deyell M.W., Tang L., Hawkins N.M., Sedlak T., Nault I., Verma A., Khairy P., Andrade J.G. (2020). Impact of Female Sex on Clinical Presentation and Ablation Outcomes in the CIRCA-DOSE Study. JACC Clin. Electrophysiol..

[90] Du Fay De Lavallaz J., Clerc O., Pudenz C., Illigens B., Kühne M. (2019). Sex-specific Efficacy and Safety of Cryoballoon versus Radiofrequency Ablation for Atrial Fibrillation: A Systematic Review and Meta-analysis. Cardiovasc. Electrophysiol..

[91] Kuck K.-H., Brugada J., Fürnkranz A., Chun K.R.J., Metzner A., Ouyang F., Schlüter M., Elvan A., Braegelmann K.M., Kueffer F.J. (2018). Impact of Female Sex on Clinical Outcomes in the FIRE AND ICE Trial of Catheter Ablation for Atrial Fibrillation. Circ. Arrhythmia Electrophysiol..

[92] Turagam M.K., Neuzil P., Schmidt B., Reichlin T., Neven K., Metzner A., Hansen J., Blaauw Y., Maury P., Arentz T. (2023). Clinical Outcomes by Sex After Pulsed Field Ablation of Atrial Fibrillation. JAMA Cardiol..

[93] Pancholy S.B., Sharma P.S., Pancholy D.S., Patel T.M., Callans D.J., Marchlinski F.E. (2014). Meta-Analysis of Gender Differences in Residual Stroke Risk and Major Bleeding in Patients with Nonvalvular Atrial Fibrillation Treated with Oral Anticoagulants. Am. J. Cardiol..

[94] Curcio A., Anselmino M., Di Biase L., Migliore F., Nigro G., Rapacciuolo A., Sergi D., Tomasi L., Pedrinelli R., Mercuro G. (2023). The Gray Areas of Oral Anticoagulation for Prevention of Thromboembolic Events in Atrial Fibrillation Patients. J. Cardiovasc. Med..

[95] Yong C.M., Tremmel J.A., Lansberg M.G., Fan J., Askari M., Turakhia M.P. (2020). Sex Differences in Oral Anticoagulation and Outcomes of Stroke and Intracranial Bleeding in Newly Diagnosed Atrial Fibrillation. JAHA.

[96] Weberndörfer V., Beinart R., Ricciardi D., Ector J., Mahfoud M., Szeplaki G., Hemels M., DAS-CAM participants 2017/2018 (2019). Sex Differences in Rate and Rhythm Control for Atrial Fibrillation. EP Eur..

[97] Russo A.M., Zeitler E.P., Giczewska A., Silverstein A.P., Al-Khalidi H.R., Cha Y.-M., Monahan K.H., Bahnson T.D., Mark D.B., Packer D.L. (2021). Association Between Sex and Treatment Outcomes of Atrial Fibrillation Ablation Versus Drug Therapy: Results from the CABANA Trial. Circulation.

[98] Henry L., Hunt S., Holmes S.D., Martin L.M., Ad N. (2013). Are There Gender Differences in Outcomes after the Cox-Maze Procedure for Atrial Fibrillation?. Innovations.

[99] Linde C., Bongiorni M.G., Birgersdotter-Green U., Curtis A.B., Deisenhofer I., Furokawa T., Gillis A.M., Haugaa K.H., Lip G.Y.H., Van Gelder I. (2018). Sex Differences in Cardiac Arrhythmia: A Consensus Document of the European Heart Rhythm Association, Endorsed by the Heart Rhythm Society and Asia Pacific Heart Rhythm Society. EP Eur..

[100] Tsai W.-C., Haung Y.-B., Kuo H.-F., Tang W.-H., Hsu P.-C., Su H.-M., Lin T.-H., Chu C.-S., Jhuo S.-J., Lee K.-T. (2016). Hormone Replacement Therapy and Risk of Atrial Fibrillation in Taiwanese Menopause Women: A Nationwide Cohort Study. Sci. Rep..

[101] Apostolakis S., Sullivan R.M., Olshansky B., Lip G.Y.H. (2014). Hormone Replacement Therapy and Adverse Outcomes in Women with Atrial Fibrillation: An Analysis from the Atrial Fibrillation Follow-Up Investigation of Rhythm Management Trial. Stroke.

[102] Blackwell K., Blackwell M., Blackwell T. (2023). Testosterone Replacement Therapy and Cardiovascular Disease: Balancing Safety and Risks in Hypogonadal Men. Curr. Cardiol. Rep..

[103] Olié V., Canonico M., Scarabin P.-Y. (2011). Postmenopausal Hormone Therapy and Venous Thromboembolism. Thromb. Res..

[104] Marzak H., Ringele R., Matsushita K., Marchandot B., Fitouchi S., Cardi T., Kanso M., Schatz A., Hammann J., Ohlmann P. (2023). Impact of Gender on Left Atrial Low-Voltage Zones in Patients with Persistent Atrial Fibrillation: Results of a Voltage-Guided Ablation. Front. Cardiovasc. Med..

[105] Park H., Kwon O.-S., Shim J., Kim D., Park J.-W., Kim Y.-G., Yu H.T., Kim T.-H., Uhm J.-S., Choi J.-I. (2025). Artificial Intelligence-Estimated Electrocardiographic Sex as a Recurrence Predictor after Atrial Fibrillation Catheter Ablation. Eur. Heart J.-Digit. Health.

[106] Brunetti N.D., Curcio A., Nodari S., Parati G., Carugo S., Molinari M., Acquistapace F., Gensini G., Molinari G., Working Group on Telecardiology, Informatics of the Italian Society of Cardiology (2023). The Italian Society of Cardiology and Working Group on Telecardiology and Informatics 2023 Updated Position Paper on Telemedicine and Artificial Intelligence in Cardiovascular Disease. J. Cardiovasc. Med..

[107] Curcio A., Quarta R. (2024). Transesophageal Echocardiography before Atrial Fibrillation Ablation: To Do or Not to Do?. Pol. Heart J..

[108] Kumagai K., Nakashima H., Urata H., Gondo N., Arakawa K., Saku K. (2003). Effects of Angiotensin II Type 1 Receptor Antagonist on Electrical and Structural Remodeling in Atrial Fibrillation. J. Am. Coll. Cardiol..

[109] Karakasis P., Patoulias D., Popovic D.S., Pamporis K., Theofilis P., Nasoufidou A., Stachteas P., Samaras A., Tzikas A., Giannakoulas G. (2024). Effects of Mineralocorticoid Receptor Antagonists on New-Onset or Recurrent Atrial Fibrillation: A Bayesian and Frequentist Network Meta-Analysis of Randomized Trials. Curr. Probl. Cardiol..

[110] Paw M., Kusiak A.A., Nit K., Litewka J.J., Piejko M., Wnuk D., Sarna M., Fic K., Stopa K.B., Hammad R. (2023). Hypoxia Enhances Anti-Fibrotic Properties of Extracellular Vesicles Derived from hiPSCs via the miR302b-3p/TGFβ/SMAD2 Axis. BMC Med..

[111] Mohammad Z., Ahmad J., Sultan A., Penagaluri A., Morin D., Dominic P. (2023). Effect of Sacubitril–Valsartan on the Incidence of Atrial Fibrillation: A Meta-analysis. Cardiovasc. Electrophysiol..

[112] Karakasis P., Fragakis N., Patoulias D., Theofilis P., Kassimis G., Karamitsos T., El-Tanani M., Rizzo M. (2024). Effects of Glucagon-Like Peptide 1 Receptor Agonists on Atrial Fibrillation Recurrence After Catheter Ablation: A Systematic Review and Meta-Analysis. Adv. Ther..

[113] Yamaguchi S., Maeda M., Oba K., Maimaituxun G., Arasaki O., Yagi S., Kusunose K., Soeki T., Yamada H., Fukuda D. (2024). Sex Differences in the Association between Epicardial Adipose Tissue Volume and Left Atrial Volume Index. BMC Cardiovasc. Disord..

[114] Kidess G.G., Hamza M., Goru R., Basit J., Alraiyes M., Alraies M.C. (2025). The Impact of Sodium-Glucose Cotransporter-2 Inhibitors on Atrial Fibrillation Burden in Diabetic Patients. Am. J. Cardiol..

[115] Bikou O., Thomas D., Trappe K., Lugenbiel P., Kelemen K., Koch M., Soucek R., Voss F., Becker R., Katus H.A. (2011). Connexin 43 Gene Therapy Prevents Persistent Atrial Fibrillation in a Porcine Model. Cardiovasc. Res..

